# Attention focused on memory: The episodic flanker effect with letters, words, colors, and pictures

**DOI:** 10.3758/s13414-024-02965-9

**Published:** 2024-10-09

**Authors:** Gordon D. Logan, Keanani C. S. Afu, Bailey E. Haynes, Ella E. Weeks, Jana E. Ulrich, Simon D. Lilburn

**Affiliations:** Department of Psychology, Vanderbilt University, 2201 West End Ave, Nashville, TN 37235 USA

**Keywords:** Visual memory, Verbal memory, Serial memory, Attention and memory, Flanker interference effects

## Abstract

We report 10 experiments exploring the proposition that memory retrieval is perceptual attention turned inward. The experiments adapt the Eriksen and Eriksen perceptual flanker effect to a memory task in which subjects must decide whether a cued item in a probe display appeared in the same position in a memory list. Previous research with this *episodic flanker task* found distance and compatibility effects like those in the perceptual flanker task, suggesting that the same attentional spotlight is turned inward in memory retrieval. The previous experiments used lists of six consonants. The experiments reported here were designed to generalize the results to a broader range of conditions, from letters to words, colors, and pictures, and from set size 6 to set sizes of 4 and 5. Experiments 1–4 varied distance and set size with lists of four, five, or six letters, words, colors, and pictures, respectively. The distance effect was observed with all materials and all set sizes. Experiments 5–8 varied compatibility by presenting context items in the probe that were either the same as the memory list (and therefore compatible with “yes” responses and incompatible with “no” responses) or different from the memory list (and therefore incompatible with “yes” responses and compatible with “no” responses). We found compatibility effects with all materials and all set sizes. These results support the proposition that memory retrieval is attention turned inward. Turned inward or outward, attention is a general process that applies the same computations to different kinds of materials.

## Introduction

Perceptual selection and memory retrieval solve similar computational problems. Finding the third book on the top shelf of your bookcase is like remembering what you had for dinner on the third day of your vacation. In both cases, target information must be extracted from a complex multidimensional structure, using the structure to guide extraction. We propose that the two problems are solved by the same computational mechanism: selective attention. Turned outward, it selects information from perception. Turned inward, it selects information from memory. Memory retrieval is selective attention turned inward (Logan et al., 2021). We are not the first to consider the relations between attention and memory (e.g., Broadbent, 1957; Cowan, 1988; Craik & Tulving, 1975; James, 1890; Nobre et al., 2004; Norman, 1968; Rugg et al., 2008), but we offer a new perspective, a new task, and new models aimed at integrating them empirically and theoretically.

We addressed the proposal empirically by adapting the famous C. W. Eriksen and Hoffman (1972) and B. A. Eriksen and Eriksen (1974) *perceptual flanker task* to memory retrieval in an *episodic flanker task* that requires focusing attention on a single item in memory. The episodic flanker task shows distance and compatibility effects like the perceptual flanker task (B. A. Eriksen & Eriksen, 1974; Logan et al., 2021) and similar benefits from precuing target location (C. W. Eriksen & Hoffman, 1972; Logan et al., 2023a, 2023b), suggesting that the same computational mechanism implements selection in memory and perception. We addressed the proposal computationally by applying quantitative models of serial recall to the episodic flanker task, interpreting their retrieval cues as spotlights of attention focused on memory. The models fit well, showing that the retrieval mechanisms produced effects identified with selective attention in the perceptual flanker task (Logan et al., 2021).

The goal of the present research is to assess the generality of distance and compatibility effects in the episodic flanker task and the conclusions we drew from them. We believe the same mechanism of attention is at work in all cases of memory retrieval. We test our belief with 10 experiments. Our research so far has focused on retrieval from lists of six random consonants. Here, we attempt to replicate distance and compatibility effects with lists of four, five, and six consonants, words, colors, and pictures, spanning a broader range of list lengths and materials that are more representative of the memory literature.

## The perceptual flanker task

The perceptual flanker task was developed in the late 1960s and early 1970s by Charles Eriksen and his colleagues, who published much of their work in this journal. Eriksen was intrigued by the Averbach and Coriell (1961) version of the Sperling (1960) partial report task, which used a bar cue below a string of letters to probe memory at different delays after the letter string was extinguished (subjects had to report the letter next to the bar). Eriksen realized he could study the time course of focusing attention by presenting the bar cue before the letters. He and his colleagues showed that increasing precue delay benefitted response time (RT) and accuracy (Eriksen & Collins, 1969; Eriksen & Hoffman, 1972, 1973). Eriksen and Hoffman (1972, 1973) presented letters flanking the cued letter and manipulated the distance and the compatibility between them (compatible letters were mapped to the same response; incompatible letters were mapped to opposite responses). They found shorter RT and lower error rate with greater distances and compatible flankers. Eriksen and Eriksen (1974) removed the requirement to search the display for the cued position, presenting the letters in a row in the center of the screen and asking subjects to classify the central letter (e.g., displays: HHSHH, SSSSS; task: “Is the central letter an H or an S?”). They manipulated compatibility (HHHHH is compatible; HHSHH is incompatible) and distance between the target and the flankers (HHSHH is near; HH S HH is far) to measure the breadth of the focus of attention, on the hypothesis that letters that fall within the spotlight of attention (near displays) will be processed and influence RT and error rate but letters that fall outside it (far displays) will not. They found robust compatibility and distance effects that have replicated hundreds of times, making the perceptual flanker task a gold-standard method for studying focused attention (Eriksen, 1995). It has been observed with a wide range of materials (Fan et al., 2002; Ridderinkhof et al., 2021; Rueda et al., 2004; Servant et al., 2014; Shaffer & LaBerge, 1979; Snell & Grainger, 2018), and it is a popular target for theorizing (Hübner et al., 2010; Lee & Sewell, 2024; Logan, 1996; Ulrich et al., 2015; White et al., 2011).

## The episodic flanker task

The episodic flanker task was designed to implement the perceptual flanker task with attention turned inward on memory. Subjects were given a row of six letters to remember (e.g., ABCDEF) followed by a probe containing a position cue and a letter, and they were asked whether the cued letter occupied the same position in the memory list (e.g., ##C### requires a “yes” response, where the underline represents a caret ^ cue presented under the cued position). To enforce attention to the cued position on the list, we presented lures from uncued positions, so mere presence in the list did not discriminate targets from lures (C. W. Eriksen & Hoffman, 1972, 1973). To reach a decision, subjects must focus on the cued position in the memory list and compare the letter in that position with the letter in the probe.

We asked how sharply subjects could focus attention on the cued item in memory by manipulating distance between the position of the cue and the position of the probed letter in the list. We presented lures from one or two (or more) positions away from the cued position (given list ABCDEF, ##D### is Distance 1; ##A### is Distance − 2; both require a “no” response). The distance manipulation varies the similarity between the letter in the probe and the information available at the cued location in the memory list. Letters (flankers) from nearby positions in the list are likely to fall within the spotlight and (partially) match the probe letter, providing evidence for a “yes” response, which will slow the required “no” response. Letters (flankers) from more distant positions are less likely to fall within the spotlight. They will match the probe letter less and provide less evidence for a “yes” response, which slows the required “no” response less. This analysis predicts the distance effect: “no” RT and error rate should decrease as flanker distance increases (for formal derivations of this prediction, see Logan et al., 2021). We found robust distance effects in 13 experiments that varied distance by itself (Logan et al., 2021, 2023a, 2023b, 2024) and in six experiments that varied distance and compatibility (Logan et al., 2021, 2023a, 2023b). RT was shorter and error rate was lower the greater the distance in the list, suggesting a sharp focus on the cued position.

We asked whether people could ignore distraction from flanking items by presenting letters in the uncued positions in the probe and manipulating their compatibility with the memory list and judgment (yes or no). The letters could be the *same* as the memory list (e.g., list ABCDEF; “yes” probe ABCDEF; “no” probe ABCEDF) or *different* from the memory list (e.g., list ABCDEF; “yes” probe STVDKL; “no” probe STVEKL). The flanking letters in *same* probes match the letters in the list and so provide evidence for a “yes” response that speeds performance on “yes” trials and slows performance on “no” trials. The flanking letters in *different* probes mismatch the letters in the list and so provide evidence for a “no” response that slows performance on “yes” trials and speeds performance on “no” trials. “Yes” same and “no” different trials are compatible; “yes” different and “no” same trials are incompatible. We found robust compatibility effects in six experiments (Logan et al., 2021, 2023a, 2023b). RT was shorter and error rate was lower on compatible trials. The episodic flanker task produced distance and compatibility effects like those in the perceptual flanker task, so we concluded that memory retrieval was perceptual attention turned inward, accomplished by the same computational mechanism.

These flanker effects are *episodic* because they are driven by unique stimuli that occur in a specific time and place, creating a specific event in the subject’s experience that is committed to memory (Tulving, 1972). Perceptual flanker effects are not episodic in this sense. They are often driven by a small set of stimuli that are repeated throughout the experiment (e.g., B. A. Eriksen & Eriksen, 1974) or by previously established semantic associations (Shaffer & LaBerge, 1979; Snell & Grainger, 2018). In principle, episodic flanker effects should occur in all episodic memory tasks, if they rely on memory structures that allow meaningful measures of distance and compatibility. The present experiments test that principle in serial memory tasks using different kinds of lists.

## Generalization

The conclusion that memory retrieval is attention turned inward is very broad. It requires a broader test than we provided. All our experiments used memory lists of six random consonants, following precedents in research on serial memory (Logan, 2021). Here, we report experiments that test whether the conclusions generalize to other materials and other set sizes. Generalization across materials is important. Attention is a central process that provides selective access to all sorts of perceptual information. If the distance and compatibility effects in the episodic flanker task reflect attention to memory, they should generalize across materials. The perceptual flanker task generalizes from letters (B. A. Eriksen & Eriksen, 1974) to colors (Servant et al., 2014), arrows (Fan et al., 2002; Ridderinkhof et al., 2021), words (Shaffer & LaBerge, 1979; Snell & Grainger, 2018), and pictures of fish swimming left or right (Rueda et al., 2004). The episodic flanker task should show similar generality.

We ran four experiments testing the generality of distance effects and four experiments testing the generality of compatibility effects (plus two replication experiments). Each experiment within each set used different materials: letters, words, colors, or pictures. We used letters for continuity with our previous research, words to connect with the broader literature on “verbal” memory (including the literature on serial memory), and colors and pictures to connect with the broader literature on visual short-term memory. If distance and compatibility effects reflect attention turned inward, they should occur whenever attention is turned inward on a memory list, regardless of the materials.

We presented lists in a horizontal row centered on the fixation point to ensure they would be represented in a common memory structure that allows us to define distance and order. Lists of letters are often presented in this way and encoded in left-to-right order. Studies of serial recall of words usually present lists sequentially, resulting in an ordered representation. Studies of visual short-term memory for colors and pictures often present them in random positions or equally spaced on a circle. Neither approach constrains the memory structure strongly enough to define order unambiguously. People are not consistent with themselves or with others when grouping random displays (Compton & Logan, 1993, 1999), and circular displays do not constrain the start and end of the list. Linear displays are grouped unambiguously (van Oeffelen & Vos, 1983) with clear beginnings and ends and a well-defined distance metric.

We manipulated set size, using lists of four, five, or six items. Set size is a very common manipulation in studies of short-term memory, so it is important to determine whether distance and compatibility manipulations interact with it. Set size effects differ between materials (e.g., “memory span” is often larger for letters than for colors), so we chose a range of set sizes that would allow good performance for all four types of material.

## Experiments 1–4: Distance effects

**Fig. 1:**
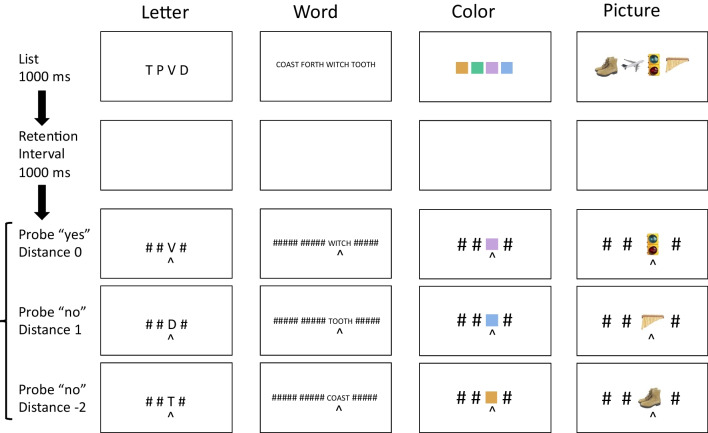
Sequence of events in Experiments 1–4. The sequence of events (top to bottom) in Experiments 1–4 (left to right) illustrating the examples of the materials used in each experiment. The displays are not drawn to scale. The spacing of the items was the same in each experiment. The bottom three rows show probe displays for lags of 0, which require a “yes” response, and lags of 1 and − 2, which require a “no” response. (Color figure online)

The first four experiments tested distance effects. The sequence of events on each trial is depicted in Fig. 1. Subjects were presented with lists of four, five, or six letters, words, colors, or pictures, followed by probes containing a single item and a position cue. Their task was to decide whether the item in the probe appeared in the cued position in the memory list. Set size was varied within subjects. The memory material was varied between subjects: letters, words, colors, and pictures were tested in separate experiments.

We manipulated distance by choosing probe items from the memory list that were − 2, − 1, 0, 1, or 2 positions away from the cued position. Distance = 0 requires a “yes” response. Distances ± 1 and ± 2 away from the cued position require a “no” response. We assessed the distance effect in “no” RTs and error rates with a planned contrast that compared distances ± 1 with distances ± 2. We used contrast weights {− 1 + 1 + 1 − 1} for distances − 2, − 1, 1, and 2, respectively. We calculated the contrast for RT and error rate for each subject and concluded there was a distance effect if the mean of the contrast values across subjects was significantly different from zero. We calculated distance contrasts for each set size and each set of materials to assess the generality of the distance effect. To assess changes in the distance effects with set size, we calculated a linear contrast evaluating the change in the distance contrast with set size.

## Method

### Subjects

Each experiment tested 32 subjects recruited online through Prolific (https://www.prolific.co/). With this sample size, we found robust distance effects in 13 experiments that varied distance by itself (Logan et al., 2021, 2023a, 2023b, 2024), and in six experiments that varied distance and compatibility (Logan et al., 2021, 2023a, 2023b). We included only subjects 18–40 years of age, located in the USA, with English as their first language, with an approval rating of at least 95, who performed with at least 60% accuracy on the episodic flanker task. We replaced two, three, two, and seven subjects in Experiments 1–4, respectively, who failed to meet the accuracy criterion. Subjects who participated in one experiment were excluded from the others. Each experiment involved a single 1.5-h session. Subjects were paid USD $12 per hour. The study was approved by the Vanderbilt University Institutional Review Board.

Subjects reported their age and gender. The mean age in years (standard deviation in brackets) of the subjects was 30.5 (4.8), 32.2 (5.8), 29.4 (6.2), and 30.7 (6.6) for Experiments 1–4, respectively. The gender distribution (men:women:prefer-not-to-say) was 22:12:0, 15:16:1, 15:17:0, and 12:19:1, for Experiments 1–4, respectively.

### Apparatus and stimuli

The experiments were conducted online on subjects’ personal computers. Subjects were instructed to use Google Chrome or Mozilla Firefox to complete the experiment. Phone and tablet users were excluded in the Prolific intake, and the experiment would not run on their browsers. The trials for each session were generated individually and sent to subjects’ computers using a custom Python backend. The experiment was controlled by JavaScript in the web browser using a custom function written to operate in jsPsych (de Leeuw, 2015). When the experiment started, subjects’ web browsers were instructed to enter full-screen mode to reduce distraction.

The memory lists were presented in a horizontal line of four, five, or six unique items, centered on the fixation point (see Fig. 1). The background of this display, and all the others, was set to mid-gray ([127, 127, 127] in 24-bit RGB values). The probe displays matched the memory lists in set size and were presented in the same positions. The probe displays presented a probe item in a cued position, indicated with a caret (^) presented underneath it. The other three, four, or five positions were filled with placeholders (# symbols; see Fig. 1). Experiment 1 used capital letters in Courier font, sampling four, five, or six from a set of 20 consonants (excluding vowels and *Y*), replicating our previous episodic flanker experiments with a variation in set size. Each letter was 100 pixels high and 60 pixels wide. The letters were spaced 120 pixels center to center. Set Sizes 4, 5, and 6 were 420, 540, and 660 pixels wide.

Experiment 2 used words. To equate the sampling probabilities across materials, we used two sets of 20 words: {BUILT CHIEF CORPS DOUBT DRIVE EIGHT FAITH GREEN HORSE MARCH MOUTH PLANE PRICE REACH SCENE SERVE SPEAK SPENT STAFF WRITE}, and {BREAK BROAD BUILD CHECK CLAIM DANCE DRINK FIGHT GLASS LEARN MEANT SHAPE SHARE SIGHT SPEED SPOKE STYLE TEETH TOUCH WORTH}. Half of the subjects received each set. The words were five letters long and presented in capitals in Courier font. They were sampled from the University of Western Australia MRC Psycholinguistic Database (https://websites.psychology.uwa.edu.au/school/MRCDatabase/uwa_mrc.htm). The mean Kucera–Francis frequencies were 109.75 (5.45) and 90.90 (6.78) for the first and second sets, respectively. The words were 34 pixels high and 102 pixels wide, spaced 120 pixels center to center (see Appendix B). Set Sizes 4, 5, and 6 were 460, 580, and 700 pixels wide.

**Fig. 2:**
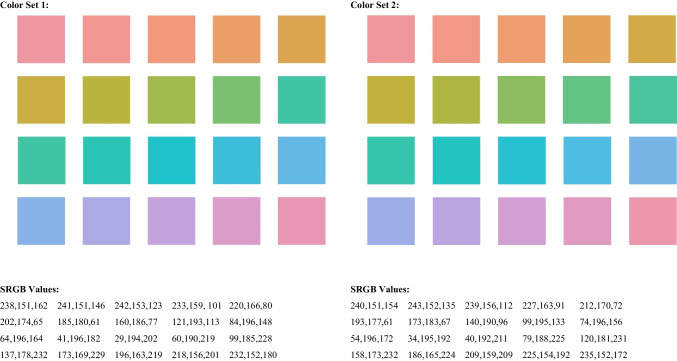
Color sets and SRGB values used in the experiments. (Color figure online)

Experiment 3 used two sets of 20 colors sampled from the Munsell renotation dataset (https://www.rit.edu/science/munsell-color-science-lab-educational-resources) (Newhall et al., 1943; see Fig. 2). They were selected to have the maximum chroma value (C = 8) for a lightness value of V = 7, which would make them equiluminant with a properly calibrated display. The colors were presented as square patches 100 × 100 pixels with a 1-pixel white outline to help define the color. They were spaced 120 pixels apart center to center. Set Sizes 4, 5, and 6 were 460, 580, and 700 pixels wide. To help distinguish the colors, lists were constructed so that colors in adjacent positions in the memory list were at least two steps apart in hue space (see Figure 2).

**Fig. 3:**
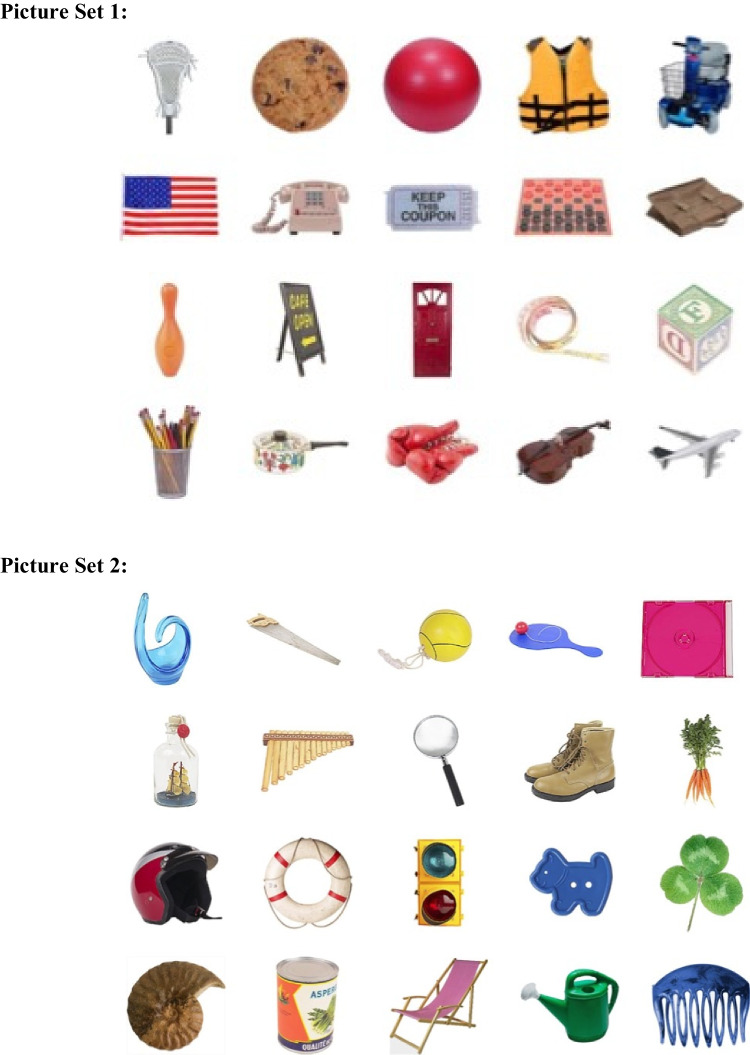
Pictures sets used in the experiments. (Color figure online)

Experiment 4 used two sets of 20 pictures sampled from the Brady et al. (2008) data set (https://bradylab.ucsd.edu/stimuli.html) (see Fig. 3). They were presented in 100 × 100 pixel regions, spaced 120 pixels apart center to center. Set Sizes 4, 5, and 6 were 460, 580, and 700 pixels wide (see Figure 3).

### Procedure

In all four experiments, each trial began with a fixation cross presented in the center of the screen for 1,000 ms. Then, the memory list was presented for 1,000 ms, followed by a blank screen for 1,000 ms, and then a probe display containing an item from the display (at Lag − 2, − 1, 0, 1 or 2), and 3, 4, or 5 # placeholders (see Fig. 1) was presented. Subjects were required to decide whether the probe item appeared in the cued position in the memory list, pressing M (or Z, counterbalanced) to indicate “yes” and Z (or M) to indicate “no.” Lag 0 trials required a “yes” response and Lag ± 1 and ± 2 trials required a “no” response. After subjects’ response, the screen went blank for a 1,000-ms intertrial interval. There were 704 trials in each experiment. Breaks were given every 64 trials. The basic design required eight trials for each cue position (four Lag 0 and one each of Lag − 2, − 1, 1, 2), so 32, 40, and 48 trials were required to balance lag and cue position for Set Sizes 4, 5, and 6. We included seven replications of the basic design for Set Size 4 for a total of 224 trials; six replications of the basic design for Set Size 5 for a total of 240 trials; and five replications of the basic design for Set Size 6 for a total of 240 trials. Set size and required response (“yes” or “no”) varied randomly from trial to trial. A separate random order was prepared for each subject.

The instructions were written and presented using a self-paced series of manually controlled slides. Subjects were allowed to review the instructions if they wished. At the end of each block, a screen was presented indicating the overall accuracy for the preceding block, and subjects were allowed to take a self-timed break. Every 5 min, the experiment checked whether accuracy was greater than 60%. If subjects fell below this criterion, they were warned to improve performance and given an opportunity to review the instructions. On the third warning, subjects were excluded from the experiment but were paid nevertheless.

### Data analysis

The purpose of Experiments 1–4 was to determine whether distance effects in the episodic flanker task generalize across materials and set size. We addressed these questions with planned linear contrasts of the form ${\sum }_{i=1}^{N}{c}_{i}{M}_{i}$ such that ${\sum }_{i=1}^{N}{c}_{i}=0$, where *N* is the number of conditions, *c*_*i*_ are the weights on means 1 to *N* and *M*_*i*_ are the means. Here and elsewhere we describe the contrast as a set of weights and the set of conditions they apply to. We assessed the distance effect in RT and error rate at each set size with a contrast using weights {− 1 + 1 + 1 − 1} for lags {− 2 − 1 1 2}. We asked whether distance effects changed with set size by assessing the linear trend relating the distance contrast to set size, using weights {− 1 0 1} for set sizes {4 5 6}. We calculated the contrasts for mean RTs and error rates for each subject and used a *t* test to determine whether the contrasts were significantly different from zero. These contrasts focus directly on the theoretically relevant questions. The raw data and the means for each subject in each condition of each experiment are available on the Open Science Framework (https://osf.io/dwku4/) for readers interested in other analyses.

Because the effects of interest are planned contrasts that compare conditions to assess patterns in the data, we could not construct error bars for the contrasts around the individual mean RTs and error rates that would permit statistical inferences about the pattern. Consequently, there are no error bars in our figures.

## Results and discussion

**Fig. 4:**
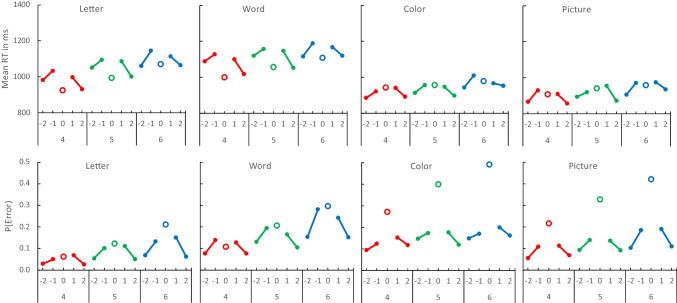
Distance effects. Mean response time (RT; top row) and error rate (P(Error); bottom row) in Experiments 1–4 (columns) as a function of set size (4, 5, 6) and distance (− 2, − 1, 0, 1, 2). Set sizes 4, 5, and 6 are presented in different colors (red, green, blue, respectively) so “yes” (lag 0) and “no” (lag ± 1 and lag ± 2) are grouped together. The distance effect is the difference between ± 1 and ± 2. (Color figure online)

The mean RTs for correct responses and error rates in each experiment are plotted as a function of distance and set size in Fig. 4. There were some quantitative differences between experiments, but each one showed significant increases in RT and error rate with set size, consistent with many previous experiments. We assessed set size effects with contrasts evaluating the significance of the linear trend. The results are reported in Table 1.

**Table 1:** Contrasts assessing linear increases in overall response time (RT) and error rate (Error) as a function of set size in Experiments 1–4

Measure	RT				P(Error)			
Experiment	1.Letter	2.Word	3.Color	4.Picture	1.Letter	2.Word	3.Color	4.Picture
*t*(31)	6.6662	3.8246	5.9737	5.2414	9.4609	14.9627	11.6187	16.5595
*SEM*	14.7060	17.6646	9.9990	10.1863	0.0110	0.0095	0.0114	0.0080
*p*	< .0001	= .0006	< .0001	< .0001	< .0001	< .0001	< .0001	< .0001
*BF*_*10*_	> 10,000	51.7415	> 10,000	1956.95	> 10,000	> 10,000	> 10,000	> 10,000

Data are averaged over “yes” and “no” responses

The novel results are the distance effects, which also appeared in the RT and error data in each experiment. RT was longer and error rate was higher for probes immediately adjacent to the cued position (± 1) than for probes two positions away (± 2), suggesting a sharp focus on the memory representation of the cued position (B. A. Eriksen & Eriksen, 1974; C. W. Eriksen & Hoffman, 1972; Logan et al., 2021, 2023a, 2023b).

These conclusions were supported by distance contrasts performed on the RTs and error rates. The contrasts {− 1 + 1 + 1 − 1} comparing positions − 2, − 1, 1, and 2 are presented in Table 2. They were significantly greater than zero for RT and error rate in each experiment except for RT for words at Set Size 5. We assessed changes in the distance effect with set size by calculating a linear contrast relating the distance contrast to set size (i.e., {− 1 0 + 1} for Set Sizes 4, 5, and 6, respectively). The linear contrast was not significant for RT in any experiment, indicating no change in the distance effect with set size. The linear contrast was significant for error rate in all experiments, indicating an increase in the distance effect with set size.

**Table 2:** Contrasts evaluating distance effects in response time (RT) and error rate (Error) at each set size in Experiments 1–4 (Letter, Word, Color, Picture) and contrasts evaluating the linear interaction between set size and distance (SS × D)

	RT				Error			
Set size	4	5	6	SS × D	4	5	6	SS × D
1. Letter								
*t*(31)	5.8839	7.7453	7.2731	0.5768	4.5007	4.0540	5.7296	3.1086
*SEM*	10.0340	8.2562	9.1355	6.4183	0.0069	0.0134	0.0134	0.0073
*p*	0.0000	0.0000	0.0000	0.5682	0.0001	0.0003	0.0000	0.0040
*BF*_*10*_	> 1000	> 1000	> 1000	.2203	283.53	91.26	> 1000	9.65
2. Word								
*t*(31)	3.5835	1.5728	5.4106	1.0653	3.3999	4.4997	4.8226	2.0854
*SEM*	13.1459	12.2211	12.2494	8.9971	0.0112	0.0168	0.0171	0.0454
*p*	0.0011	0.1259	0.0000	0.2950	0.0019	0.0001	0.0000	0.0106
*BF*_*10*_	28.89	0.57	> 1000	0.3174	18.74	282.81	652.74	1.26
3. Color								
*t*(31)	2.8019	3.1715	2.9403	− 0.1045	2.5670	3.8608	2.2051	-0.1042
*SEM*	14.9304	14.4437	13.7301	6.9955	0.0124	0.0108	0.0135	0.0101
*p*	0.0087	0.0034	0.0061	0.9174	0.0153	0.0005	0.0350	0.9177
*BF*_*10*_	4.97	11.11	6.67	0.19	3.08	56.54	1.56	0.19
4. Picture								
*t*(31)	4.9045	4.7844	4.1582	− 0.3128	4.9233	3.2207	5.7665	2.0561
*SEM*	11.8623	11.5415	12.6705	8.7796	0.0099	0.0139	0.0139	0.0077
*p*	0.0000	0.0000	0.0002	0.7565	0.0000	0.0030	0.0000	0.0483
*BF*_*10*_	808.32	590.91	118.53	0.20	849.04	12.41	> 1000	1.20

These experiments establish that the distance component of the episodic flanker effect replicates across a broad range of materials: letters, words, colors, and pictures. They suggest that our theoretical interpretation of the effect as a measure of the sharpness of the focus of attention on a memory representation generalizes across materials. Retrieving an item from a cued position in a list requires focusing attention in the same way whether the items are letters, words, colors, or pictures.

These experiments also establish that the distance component of the episodic flanker effect replicates across a range of set sizes commonly used in studies of short-term memory. Studies of colors and pictures typically use smaller set sizes (four or five) while studies of letters and words typically use larger set sizes (six or more). Finding distance effects at each set size is consistent with our theoretical interpretation of the effect as a measure of focusing attention on the memory list. The episodic flanker task requires focusing attention on a single item regardless of set size. The relative invariance of the distance effect over set size in RT and the consistent increase in the distance effect over set size in error rate remains a puzzle for future research.

## Experiments 5–8: Compatibility effects

**Fig. 5:**
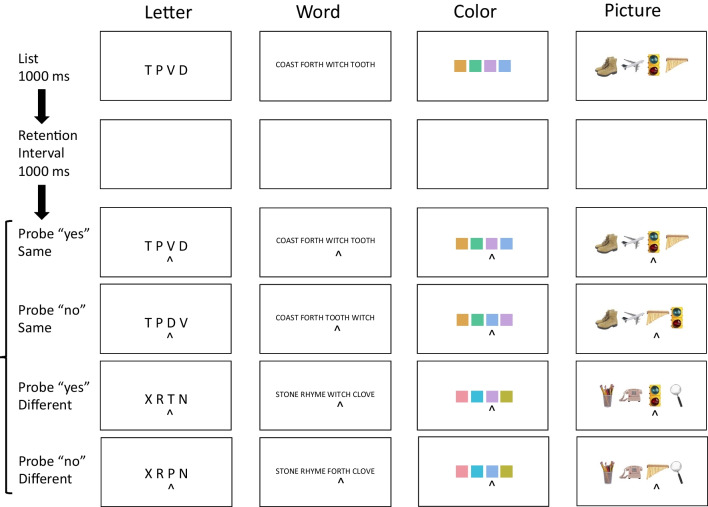
The sequence of events (top to bottom) in Experiments 5–8 (left to right) illustrating the examples of the materials used in each experiment. The displays are not drawn to scale. The spacing of the items was the same in each experiment. The bottom four rows show probe displays for the compatibility manipulation. Rows 3 and 4 illustrate probes in which the flanking items are the *same* as those in the memory list, requiring “yes” (Row 3) and “no” (Row 4) responses to the cued item. Rows 5 and 6 illustrate probes in which the flanking items are *different* from those in the memory list, requiring “yes” (Row 5) and “no” (Row 6) responses. “Yes” responses to same-context probes and “no” responses to different-context probes are compatible. “No” responses to same-context probes and “yes” responses to different-context probes are incompatible. (Color figure online)

We ran a second set of four experiments to test for compatibility effects. The timing and sequence of events on each trial is presented in Fig. 5. Subjects were presented with lists of four, five, or six letters, words, colors, or pictures followed by probes containing a context of three, four, or five items in addition to the probe item. The contexts were either the same as or different from the items in the memory list (e.g., list STVDPL; same probe STVDPL, different probe JKVCXR, where the underline represents the position cue). One position was cued with a caret (^) underneath it. It was sampled from the list at distances of − 1, 0, and 1 from the cued position. The task was to decide whether the item in the probe appeared in the cued position in the memory list. Set size was varied within subjects. The memory material was varied between subjects: letters, words, colors, and pictures were used in separate experiments.

We manipulated compatibility by presenting requiring “yes” and “no” responses to probes with same or different contexts. Same contexts match the memory list and the match provides evidence for a “yes” response. This evidence is *compatible* with probes that require a “yes” response (list STVDPL; probe STVDPL), reducing RT and error rate. It is *incompatible* with probes that require a “no” response (list STVDPL; same probe STDVPL), increasing RT and error rate. Different contexts mismatch the memory list and provide evidence for a “no” response. This evidence is *incompatible* with probes that require a “yes” response (list STVDPL; probe JLVCXR) and *compatible* with probes that require a “no” response (list STVDPL; probe JLDCXR). Altogether, these effects predict a crossover interaction between context (same, different) and response type (“yes,” “no”).

We assessed the compatibility effect with a planned contrast that evaluated the crossover interaction. We used contrast weights {+ 1 − 1 − 1 + 1} for same-context “yes,” same-context “no,” different-context “yes,” and different-context “no” trials, respectively. We calculated the contrast separately for the RT and error data from each subject and asked whether the mean contrast across subjects was significantly different from zero. To assess generalization of the compatibility effect, we calculated the contrast for each set size with each type of materials. We assessed changes in the compatibility effect with set size with a contrast using weights {− 1 0 1} for set sizes 4, 5, and 6 to evaluate the linear relation between the compatibility contrast and set size.

## Method

### **Subjects**

Each experiment tested 32 subjects recruited online through Prolific (https://www.prolific.co/) using the same inclusion criteria and payment schedule as in Experiments 1–4. With this sample size, we found robust compatibility effects in six experiments (Logan et al., 2021, 2023a, 2023b). We replaced five, two, 15, and five subjects who failed to meet the 60% accuracy criterion in the episodic flanker task in Experiments 5–8, respectively. Subjects found the color task exceptionally difficult. We decided not to make it easier to maintain continuity with Experiment 3. Subjects reported their age and gender. The mean age in years (standard deviation in brackets) of the subjects was 32.0 (6.1), 31.9 (4.9), 30.3 (6.0), and 29.5 (5.6) for Experiments 5–8, respectively. The gender distribution (men:women:prefer-not-to-say) was 18:14:0, 17:15:0, 12:20:0, and 14:18:0, for Experiments 1–4, respectively.

### Apparatus and stimuli

Experiments 5–8 were run online with the same internet protocol, the same spacing, and the same timing as Experiments 1–4 (see Fig. 5). The materials (letters, words, colors, and pictures) and the positioning and spacing of list and probe displays were the same as in Experiments 1–4. The items in the probe displays differed. The uncued positions contained items of the same type instead of fillers (#), and the items were either the *same* as or *different* from the current memory list (see Fig. 5). For displays requiring a “yes” response, the cued position in the probe contained an item that matched the item in the corresponding position in the previous list, as in the previous experiments. For displays requiring a “no” response, the cued position in the probe contained an item that was immediately before or after the cued position in the memory list (i.e., Lag − 1 or + 1).

### Procedure

The timing and sequence of events in Experiments 5–8 were the same as in Experiments 1–4. The only difference was the probe displays, which contained other items of the same type in uncued positions (see Fig. 5). As before, subjects had to press the Z or M key to indicate whether the cued item in the probe appeared in the cued position in the list. The basic 2 (“yes” vs. “no” response) × 2 (context *same* or *different*) design required four trials. Balancing this design with cue position required 16, 20, and 24 trials for Set Sizes 4, 5, and 6, respectively. We included 14, 12, and 10 replications of these sets of trials to produce 224, 240, and 240 trials for Set Sizes 4, 5, and 6, respectively. There were 704 trials in total. Breaks were allowed every 64 trials.

### Data analysis

The purpose of Experiments 5–8 was to determine whether compatibility effects in the episodic flanker task generalize across materials and set size. We addressed these questions with planned contrasts. We assessed the compatibility effect in RT and error rate with a contrast using weights {+ 1 − 1 − 1 + 1} for same-context “yes,” same-context “no,” different-context “yes,” and different-context “no” trials, respectively. We asked whether compatibility effects changed with set size by assessing the linear trend relating the compatibility contrast to set size, using weights {− 1 0 1} for Set Sizes 4, 5, and 6. We calculated the contrasts for mean RTs and error rates for each subject and used a *t* test to determine whether the contrasts were significantly different from zero. These contrasts focus directly on the theoretically relevant questions. The raw data and the means for each subject in each condition of each experiment are available on the Open Science Framework (https://osf.io/dwku4/) for readers interested in other analyses.

## Results and discussion

**Fig. 6:**
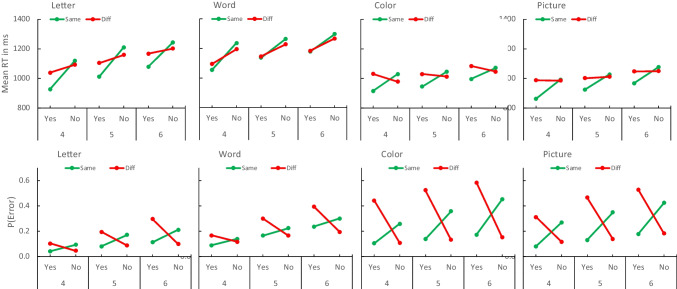
Mean response time (RT; top row) and error rate (P(Error); bottom row) in Experiments 5-8 (columns) as a function of set size (4, 5, 6), required response (Yes, No) and probe context (Same in green, Different in red). The compatibility effect is the crossover interaction between required response and probe context. (Color figure online)

The mean RTs for correct responses and error rates in each experiment are plotted as a function of set size and the compatibility interaction between response type and context in Fig. 6. As before, there were quantitative differences but broad similarities among the experiments. RT and error rate increased significantly with set size with letters, words, colors, and pictures (contrasts evaluating linear trends are presented in Table 3).

**Table 3:** Contrasts assessing linear increases in overall response time (RT) and error rate (Error) as a function of set size in Experiments 5–8

Measure	RT				P(Error)			
Experiment	5.Letter	6.Word	7.Color	8.Picture	5.Letter	6.Word	7.Color	8.Picture
*t*(31)	7.9878	5.0091	5.5144	7.6151	9.9927	10.0268	8.1446	11.1701
*SEM*	16.1688	21.0003	11.0367	10.3767	0.0107	0.0092	0.0064	0.0067
*p*	< .0001	< .0001	< .0001	< .0001	< .0001	< .0001	< .0001	< .0001
*BF*_*10*_	> 10,000	1062.88	4020.67	> 10,000	> 10,000	> 10,000	> 10,000	> 10,000

Data are averaged over “yes” and “no” responses

The predicted crossover interaction indicating a compatibility effect was observed in RT and error rate at each set size in each experiment, suggesting that subjects were unable to completely avoid distraction from the flankers in each experiment (B. A. Eriksen & Eriksen, 1974; C. W. Eriksen & Hoffman, 1972; Logan et al., 2021, 2023a, 2023b). These conclusions were supported by the contrasts that assess the crossover interaction, reported in Table 4 (i.e., weights {+ 1 − 1 − 1 + 1} for same-context “yes,” same-context “no,” different-context “yes,” and different-context “no,” respectively). All the contrasts were significantly different from zero for RT and error rate. We calculated contrasts to ask whether the compatibility effect varied linearly with set size using weights {− 1 0 + 1} for the interaction contrasts for Set Sizes 4, 5, and 6, respectively. The compatibility effect did not increase significantly with set size for RT in any experiment, but it increased significantly with set size for error rate in every experiment.

**Table 4:** Contrasts evaluating compatibility effects in response time (RT) and error rate (Error) at each set size in Experiments 5–8 (Letter, Word, Color, Picture) and contrasts evaluating the linear interaction between set size and compatibility (SS × C)

	RT				Error			
Set size	4	5	6	SS × C	4	5	6	SS × C
1. Letter								
*t*(31)	8.5360	10.4825	6.1833	− 0.4972	4.9154	6.9319	8.5416	6.5171
*SEM*	16.2894	13.5711	20.8777	10.0097	0.0221	0.0286	0.0345	0.0143
*p*	0.0000	0.0000	0.0000	0.6226	0.0000	0.0000	0.0000	0.0000
*BF*_*10*_	> 1000	> 1000	> 1000	0.21	831.68	> 1000	> 1000	> 1000
2. Word								
*t*(31)	8.2324	4.8493	2.7231	2.7423	4.4675	6.1831	9.0816	9.0895
*SEM*	17.4692	21.1868	23.9010	14.3539	.0352	.0398	.0432	.0129
*p*	.0000	.0000	.0011	.0100	.0352	.0398	.0432	.0000
*BF*_*10*_	> 1000	699.81	4.22	4.39	260.35	> 1000	> 1000	> 1000
3. Color								
*t*(31)	7.6210	4.5175	3.5055	− 1.8346	11.7807	12.9784	15.0446	6.7316
*SEM*	22.0878	25.8639	32.2567	15.0595	0.0414	0.0471	0.0472	0.0165
*p*	0.0000	0.0001	0.0014	0.0762	0.0000	0.0000	0.0000	0.0000
*BF*_*10*_	> 1000	296.06	24.01	0.84	> 1000	> 1000	> 1000	> 1000
4. Picture								
*t*(31)	7.3648	4.4384	5.7502	− 1.4022	13.2443	17.5357	15.2895	6.3271
*SEM*	17.8921	21.0598	19.0777	0.1376	0.0288	0.0311	0.0387	0.0001
*p*	0.0000	0.0001	0.0000	0.1708	0.0000	0.0000	0.0000	0.0000
*BF*_*10*_	> 1000	> 1000	> 1000	0.46	> 1000	> 1000	> 1000	> 1000

These experiments establish that the compatibility component of the episodic flanker effect replicates across letters, words, colors, and pictures. They suggest our interpretation of the effect as an inability to completely resist distraction generalizes across materials. Neighboring items intrude whether we are paying attention to perceptual displays or memory lists. The intrusion depends on the structure of the list and the focus of attention. The experiments suggest it does not depend on the nature of the items.

These experiments also establish that the compatibility component of the episodic flanker effect replicates across Set Sizes 4–6, covering the typical range in short-term memory experiments. As with the distance effect in Experiments 1–4, the compatibility effect was relatively invariant with set size in RT but increased with set size in error rate. Like the dissociation in Experiments 1–4, this dissociation remains a puzzle for future research. We are encouraged by the similarities across different measures of the ability to focus attention on memory. They suggest that the same factor may be responsible for both dissociations.

## Experiments 9–10

Experiments 2 and 6 were not our first experiments with word lists. We originally ran them with lists presented vertically. To increase comparability, Experiments 2 and 6 were replications of the original experiments with lists presented horizontally with the same spacing as the letter, color, and picture experiments. Here, we report the original experiments with vertical spacing. Experiment 9 examines distance effects. Experiment 10 examines compatibility effects.

### Method

#### Subjects

Each experiment tested 32 subjects sampled from Prolific using the same exclusion criteria as the previous experiments. We replaced five subjects in Experiment 9 and six subjects in Experiment 10 for failing to meet the 60% accuracy criterion on the episodic flanker task. For Experiments 9 and 10, respectively, the mean ages in years were 29.3 (5.6) and 32.0 (6.4), and the gender distribution was 17:15:0 and 17:15:0.

#### Apparatus and stimuli

**Fig. 7:**
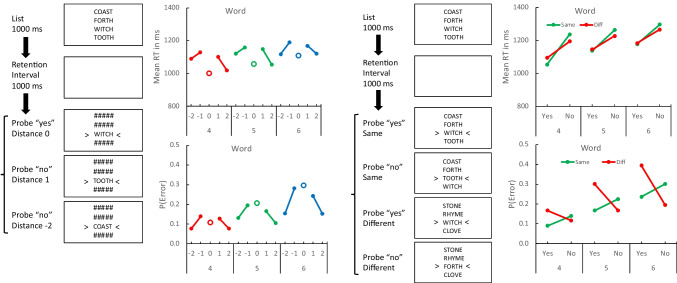
Left two columns: Sequence of events on each trial (first column) and mean response time (RT; second column top) and error rate (P(Error); second column bottom) in Experiment 9 as a function of distance and set size (4, 5, 6). The displays are not drawn to scale. The distance effect is the difference between ± 1 and ± 2. Right two columns: Sequence of events on each trial (third column) and mean RT and error rate (fourth column) in Experiment 10 as a function of the required response (Yes, No). and the probe context (Same in green, Different in red). The compatibility effect is the crossover interaction between required response and probe context. (Color figure online)

The apparatus and stimuli were the same as in Experiments 2 and 6, as were the timing and the sequence of events on each trial. Word lists were constructed in the same manner from the same sets of 20 words. They were displayed vertically rather than horizontally (see Fig. 7). Words were rendered in Courier font. They were 45 pixels high and 152 pixels wide, spaced 65 pixels apart vertically, center to center. Set Sizes 4, 5, and 6 were 240, 305, and 379 pixels high, respectively, and centered on the fixation point. The position cues were different. Instead of a single caret underneath the cued position, there were two rotated “carets” (> and <) on either side of the cued item (see Fig. 7).

#### Procedure

The procedure was the same as in Experiments 2 and 6.

### Results and discussion

The results with vertical lists and probes, presented in Fig. 7, were very similar to the previous results with horizontal lists and probes. Overall, RT and error rate increased significantly with set size in Experiment 9—for RT, *t*(31) = 5.3579, *p* < 0.0001, *SEM* = 16.0979; for error rate, *t*(31) = 15.3663, *p* < 0.0001, *SEM* = 0.0147—and Experiment 10—for RT, *t*(31) = 5.4829, *p* < 0.0001, *SEM* = 15.7909; for error rate, *t*(31) = 15.2423, *p* < 0.0001, *SEM* = 0.0101. Contrasts evaluating the distance and compatibility effects and their change with set size are reported in Table 5. Experiment 9 replicated the distance effect with vertical word lists. The distance effect was significant for RT and error rate for all set sizes. There was no evidence that the distance effect in RT changed with set size: The linear interaction between distance and set size was not significant. The distance effect in error rate was significant at all set sizes and increased significantly with set size.

**Table 5:** Contrasts for response time (RT) and error rate (Error) evaluating distance and the interaction of distance with set size (SS × D) in Experiment 9, and contrasts for RT and error rate evaluating compatibility and the interaction of compatibility with set size (SS × C) in Experiment 10

Set Size	4	5	6	SS × D/C	4	5	6	SS × D/C
9. Distance	RT				P(Error)			
*t*(31)	5.8225	5.9066	3.8410	0.0104	5.6685	5.0183	7.9212	3.0079
*SEM*	10.3115	11.0497	15.6821	9.3458	0.0098	0.0125	0.0137	0.0087
*p*	0.0000	0.0000	0.0006	0.9917	0.0000	0.0000	0.0000	0.0052
*BF*_*10*_	> 1000	> 1000	53.86	0.19	> 1000	> 1000	> 1000	7.73
10. Compatibility	RT				P(Error)			
*t*(31)	3.6816	2.2440	1.7746	− 1.7567	4.1489	5.5883	6.5085	5.1053
*SEM*	22.2764	19.7806	20.4216	13.0280	0.0241	0.0346	0.0406	0.0161
*p*	0.0009	0.0321	0.0858	0.0888	0.0002	0.0000	0.0000	0.0000
*BF*_*10*_	36.55	1.67	0.76	0.74	115.78	> 1000	> 1000	> 1000

Experiment 10 replicated the compatibility effect with vertical word lists. For RT, the critical crossover interaction between probe context and required response was significant for Set Sizes 4 and 5 but not for Set Size 6. It became significantly smaller as set size increased. For error rate, the crossover interaction was significant for all set sizes and it became significantly larger as set size increased.

## General discussion

The purpose of this article is to determine whether the distance and distraction effects in the episodic flanker task generalize beyond lists of letters to words, colors, and pictures and to determine whether they generalize beyond Set Size 6 to Set Sizes 4 and 5. The results suggest the effects generalize. Experiments 1–4 found distance effects in RT and error rate with each set of materials and each set size. The RT effect at Set Size 5 in word lists in Experiment 6 was the only exception. Experiments 5–8 found compatibility effects in RT and error rate with each set of materials and each set size. Experiments 9 and 10 replicated the word experiments with vertical lists. In Experiment 9, the distance effect in RT and error rate was significant at all set sizes. In Experiment 10, the compatibility effect was significant at all set sizes in RT and error rate except for RTs at Set Size 6. These results suggest that both the distance and distraction components of the episodic flanker effect generalize across materials (letters, words, colors, pictures) and set sizes (4, 5, 6).

We were surprised to find that distance (Experiments 1–4 and 9) and compatibility effects (Experiments 5–8 and 10) increased with set size in error rate but not in RT. Our intuitions led us to expect similar effects in the two measures. We have no coherent explanation of this finding. It emphasizes the importance of understanding the relation between attention and set size in memory retrieval. Decades of research on the relation between attention and set size in perception have been very productive, yielding hosts of important results and clarifying theory substantially. We hope the same clarity emerges in research on attention to memory.

Distance and distraction effects were common to all materials but other effects differed among them (see Figs. 4 and 6). RT was longer with letters and words than with colors and pictures, but errors—especially missed targets—were more frequent with colors and pictures. The increase in RT with set size was greater for letters and words than for colors and pictures. The increase in error rate with set size varied less systematically with materials. Our experiments focused on distance and distraction effects and so included no conditions that would allow clear interpretations of these differences, so we did not analyze them formally. The materials were not comparable. We controlled similarity within each type of material but we made no attempt to control similarity between materials. For example, pictures may be less similar to each other than colors because they vary in many more dimensions. Words may be less similar to each other than letters because they vary in semantic dimensions as well as orthographic dimensions. Differences among the materials may disappear when similarity is equated (see, e.g., Visscher et al., 2007).

The evidence that distance and distraction effects generalize from letters to words, colors, and pictures supports the broader hypothesis that memory retrieval is attention turned inward. In theory, the same attention mechanism should apply to ordinal structures regardless of the materials that are associated with them. Attention is commonly construed as a general process that can be applied to many different types of material. Distance and compatibility effects are observed in the perceptual flanker task with a wide range of materials (e.g., letters, B. A. Eriksen & Eriksen, 1974; words, Shaffer & LaBerge, 1979; Snell & Grainger, 2018; left- and right-pointing arrows, Fan et al., 2002; Ridderinkhof et al., 2021; colors, Servant et al., 2014; pictures of fish swimming left or right, Rueda et al., 2004), reflecting the sharpness of focused attention and the inability to resist distraction in each case. Our results show a similar generalization across materials in the episodic flanker task, suggesting that the same computational mechanism extracts information from perception and memory. They strengthen our claim that memory retrieval is attention turned inward (Logan et al., 2021, 2023a, 2023b).

The distance and compatibility effects reflect the operation of a retrieval process on a memory structure. Attention is focused on an element of the structure and samples information from that element and from its neighbors in the structure. The “distance” in the distance effect is defined in terms of the structure. The compatibility effect depends on correspondence between the probe and the memory list. With same-context probes, the same items appear in the same (corresponding) positions in the structure. Different-context probes present different items in those positions, destroying correspondence. Logan et al. (2021) compared same probes with scrambled probes made of the same letters assigned to random positions and found much smaller compatibility effects with scrambled probes.

The distance and compatibility effects show that structure matters. Attention must navigate the structure to find the information it is seeking. To understand the movement of attention in memory, we must understand the structure it moves through. Studies of verbal memory have addressed questions of structure enthusiastically, proposing theories of memory structures in serial recall, cued recall, free recall, and item recognition (Bower, 1967; Eichenbaum, 2017; Kintsch, 1988; Mandler, 1967; Polyn et al., 2009; Zacks & Tversky, 2001). Studies of visual memory appear to assume axiomatically that objects are structured in two- or three-dimensional (Euclidian?) space, but they do not say much about how attention operates on that representation (Logan, 1995). Our results with the episodic flanker task suggest that items made of different materials can be held in the same serially ordered memory structure and retrieved in the same way by focusing attention on an element of the structure (Logan et al., 2021).

Our results contribute to the burgeoning literature showing intimate relations between attention and memory (e.g., Broadbent, 1957; Cowan, 1988; Craik & Tulving, 1975; James, 1890; Nobre et al., 2004; Norman, 1968; Rugg et al., 2008). Our conclusion that memory retrieval is perceptual attention turned inward is consistent with many perspectives in that literature. Our claim that retrieval and attention are the same computationally is much stronger and has not received much consideration in computational models of attention or memory. The models have addressed different phenomena. Models of attention usually address situations with multiple stimuli and multiple tasks and describe the computations involved in finding a target among distractors or suppressing competition from irrelevant distractors. Models of memory address situations where these factors are controlled and reduced, often presenting one stimulus at a time (e.g., sequential presentation of the memory list).

It is tempting to think that models of attention describe the movement of the spotlight and models of memory describe what happens inside the spotlight after it has been focused. But models of attention must also explain how a decision is reached about the content that falls within the spotlight and models of memory must ultimately explain how the spotlight is focused even if the phenomena they address do not (yet) require it. There has been some convergence in modeling attention to dimensions within the spotlight in attention (Bundesen, 1990; Logan, 2002) and memory (Gillund & Shiffrin, 1984; Nosofsky, 1986; Osth et al., 2023). It would be worth extending these approaches to other aspects of attention.

The idea that attention and memory retrieval are one and the same computationally suggests that the retrieval cues in memory models may be viewed as spotlights of attention turned inward. This offers a desirable economy of mechanism: Memory theories may have already provided the mechanisms required to support attention to memory. The models already exist. Their retrieval computations just need to be interpreted differently, as attention focused on memory. We have taken this approach, using the retrieval cues in three computational models of serial memory as spotlights of attention to fit distance and compatibility effects in the episodic flanker task (Logan et al., 2021). The models make different assumptions about how order is represented, which determine the nature of the retrieval cues that access order. Order is represented by regions of space (Lee & Estes, 1977), abstract position codes (Henson, 1998), or contexts derived from neighboring items (Logan, 2021), and retrieval cues address regions of space, position codes, and contexts, respectively. All three models assume that the retrieval cues activate order representations in proportion to their similarity to the cued position and similarity decreases (approximately) exponentially with distance from the cued position. The activated order representations activate item representations, which are compared with the item representation in the probe. Nearby items will be activated more than distant ones and match the probe better, producing more evidence for a “yes” response, which results in longer RT and higher error rate for nearby probes (i.e., the distance and compatibility effects). Metaphorically, the cues are spotlights and the exponential function determines the intensity of the beam focused on the memory representation. Items that fall in the central region are processed. Items that fall outside it are not. Subjects may be able to adjust the breadth of the beam by adjusting the steepness of the exponential decline (cf. C. W. Eriksen & St. James, 1986).

All three models fit the RT and error data equally well. This was bad news from the usual perspective of competitive model fitting, where the goal is to find the best-fitting model and discard the rest. It was good news for the broader purpose of understanding the relation between attention and memory retrieval. It showed that the retrieval cues of different memory models act like spotlights of attention turned inward, and that motivates further research and model development. The key to the good fit in all three models is their assumption that retrieval depends on the similarity of memory representations to the cues, which decreases exponentially with distance from the cued position.

The idea of similarity-based retrieval is pervasive in computational models of memory (Cox & Shiffrin, 2017; Gillund & Shiffrin, 1984; Howard & Kahana, 2002; Humphreys et al., 1989; Murdock, 1982, 1993; Murnane et al., 1999; Polyn et al., 2009). Computational models often assume distributed representations: Items, contexts, and retrieval cues are represented formally as lists of features expressed as points in multidimensional space. The similarity of a retrieval cue to a context or item is (typically) an exponential function of distance in that space. Retrieval cues activate all items in memory in parallel but the activation is focused sharply on a small subset most similar to the cue, like a spotlight focused on a small region of memory. The breadth of the activated region depends on the steepness of the exponential decline, which is typically a free parameter in the models. Again, subjects may have some control over the steepness to adjust the breadth of the beam, zooming attention in and out (C. W. Eriksen & St. James, 1986; Polyn et al., 2009). Thus, the idea that memory models describe the computations involved in turning attention inward may extend beyond models of serial memory to all memory models that assume exponential similarity. It may extend beyond the episodic flanker task to the memory tasks those models explain. With the right experimental designs, attention effects may be found in all memory tasks with all sorts of materials. Our generalization from letters to words, colors, and pictures is a step in that direction.
